# Plasmonics Enhanced Smartphone Fluorescence Microscopy

**DOI:** 10.1038/s41598-017-02395-8

**Published:** 2017-05-18

**Authors:** Qingshan Wei, Guillermo Acuna, Seungkyeum Kim, Carolin Vietz, Derek Tseng, Jongjae Chae, Daniel Shir, Wei Luo, Philip Tinnefeld, Aydogan Ozcan

**Affiliations:** 1 Electrical Engineering Department, University of California, Los Angeles, Los Angeles, CA 90095 USA; 2 Bioengineering Department, University of California, Los Angeles, Los Angeles, CA 90095 USA; 3 California NanoSystems Institute (CNSI), University of California, Los Angeles, Los Angeles, CA 90095 USA; 4 Department of Chemical and Biomolecular Engineering, North Carolina State University, Raleigh, NC 27695 USA; 5 Institute for Physical & Theoretical Chemistry, Braunschweig University of Technology, Braunschweig, 38106 Germany; 6 Braunschweig Integrated Centre of Systems Biology (BRICS), Braunschweig University of Technology, Braunschweig, 38106 Germany; 7 Laboratory for Emerging Nanometrology (LENA), Braunschweig University of Technology, Braunschweig, 38106 Germany; 8 Chemical and Biomolecular Engineering Department, University of California, Los Angeles, Los Angeles, CA 90095 USA; 9 Department of Surgery, University of California, Los Angeles, Los Angeles, CA 90095 USA

## Abstract

Smartphone fluorescence microscopy has various applications in point-of-care (POC) testing and diagnostics, ranging from e.g., quantification of immunoassays, detection of microorganisms, to sensing of viruses. An important need in smartphone-based microscopy and sensing techniques is to improve the detection sensitivity to enable quantification of extremely low concentrations of target molecules. Here, we demonstrate a general strategy to enhance the detection sensitivity of a smartphone-based fluorescence microscope by using surface-enhanced fluorescence (SEF) created by a thin metal-film. In this plasmonic design, the samples are placed on a silver-coated glass slide with a thin spacer, and excited by a laser-diode from the backside through a glass hemisphere, generating surface plasmon polaritons. We optimized this mobile SEF system by tuning the metal-film thickness, spacer distance, excitation angle and polarization, and achieved ~10-fold enhancement in fluorescence intensity compared to a bare glass substrate, which enabled us to image single fluorescent particles as small as 50 nm in diameter and single quantum-dots. Furthermore, we quantified the detection limit of this platform by using DNA origami-based brightness standards, demonstrating that ~80 fluorophores per diffraction-limited spot can be readily detected by our mobile microscope, which opens up new opportunities for POC diagnostics and sensing applications in resource-limited-settings.

## Introduction

Miniaturized optical reader devices have been essential for quantifying rapid diagnostic assays and bringing some of the conventional biomedical tests from the bench or laboratory settings to the point-of-care (POC). A new generation of portable optical imaging and sensing devices has recently been fostered by the surge of smartphone, 3D printing and rapid prototyping technologies^1–6^. Such mobile platforms are centered around rapidly improving imaging, sensing and computational power of smartphones as well as the design flexibility and cost-effectiveness of rapid prototyping technologies. The global mobile phone subscription number has already reached more than 7 billion, and around 80% of these mobile phones are being used in developing countries. This forms a foundation for applying these emerging mobile imaging and sensing technologies towards various grand challenges in global health. Indeed, mobile phone enabled devices have been recently developed to diagnose infectious diseases including e.g., HIV/AIDS^7^ and malaria^8, 9^, as well as for personalized health monitoring^10–15^, tracking and prognosis of chronic diseases^16, 17^.

Fluorescence is one of the predominant detection modalities for mobile phone based diagnostic tools due to the sensitivity and specificity that it enables. Mobile fluorescence imaging devices are frequently used to read DNA or antibody-based immunoassays in various POC formats such as microarrays^18, 19^, droplets^20^, paper devices^21–23^, and microfluidic chips^24, 25^. In addition, mobile fluorescence imaging tools have been used as microscopy platforms to detect nanoscale objects and molecules including single nanoparticles^26^, viruses^26^, and single DNA strands^27^. While molecular amplification assays that are based on e.g., polymerase chain reaction (PCR) and enzyme-linked immunosorbent assay (ELISA) are able to circumvent this detection challenge by increasing the number of target molecules before the optical detection step, the development of higher sensitivity mobile optical readers is still essential to reduce the assay time and rapidly detect much lower concentrations of target fluorophores. More importantly, counting and inspection of single molecules (*e.g*., optical mapping of individual DNA strands) is an emerging POC diagnostic method, which would bring in rich diagnostic information that conventional bulk assays could not provide. However, one of the current challenges towards true molecular sensing at POC settings is the lack of advanced portable devices with a much better detection sensitivity level that can target a small number of target molecules.

Here, we present a general strategy to enhance the detection sensitivity of a mobile phone based fluorescence imaging device to less than 100 molecules per diffraction-limited spot. The principle of the signal enhancement is based on surface-enhanced fluorescence (SEF) using thin silver films. We prepared a handheld SEF microscopy device installed on a smartphone, which provided a maximum fluorescence enhancement of approximately 10-fold compared to a bare glass substrate, allowing imaging of individual 50 nm fluorescent particles as well as individual QDs. Moreover, the limit of detection was estimated to be around 80 fluorophores per diffraction-limited spot by using fluorescently labeled DNA origami structures as microscopy brightness standards. We believe that this field-portable mobile phone based SEF microscopy platform opens up various new prospects for POC sensing and molecular diagnostics in resource scarce environments.

## Methods

### Design of the smartphone-based SEF microscope

**Figure 1:**
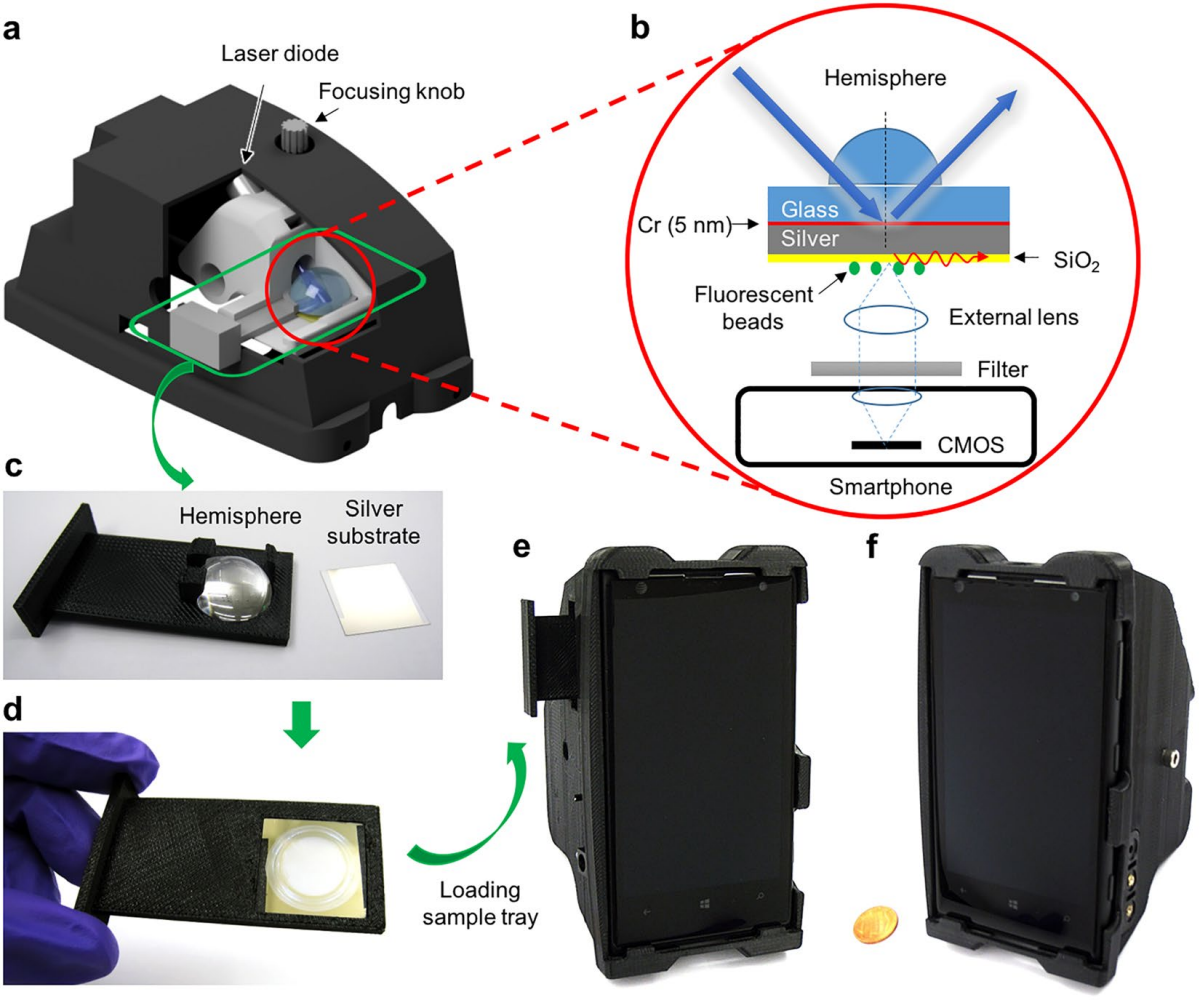
Illustrations and photographs of our mobile phone SEF microscopy device. (**a**) 3D illustration of the smartphone attachment with the cutaway view of the inner sample stage. (**b**) Schematic of the Kretschmann configuration implemented in the smartphone attachment. (**c**,**d**) Photographs of the hemisphere-embedded sample tray and silver thin film substrate, before (**c**) and after (**d**) loading of the silver-coated substrate onto the hemisphere. (**e**,**f**) Photographs of the final prototype device from different perspective views.

The smartphone-based SEF microscope attachment (see Fig. 1) was designed by Autodesk Inventor and printed by a 3D printer (Dimension Elite, Stratasys). It includes a Kretschmann illumination configuration, where a compact laser diode (465 nm, 2 W; D 12 mm × L 30 mm) excites a silver-coated glass coverslip (22 × 22 mm) through a glass hemisphere (D = 20 mm) at an illumination angle of ~58°. The laser beam is filtered by a linear polarizer (D = 10 mm, Edmund Optics) to deliver p-polarization component onto the metal film. Disposable metal coated substrates are prepared by sequential sputtering-based deposition of chromium (5 nm), silver (30 or 50 nm), and silicon dioxide (SiO_2_, 0–50 nm) on pre-cleaned glass coverslips. Both the sample tray and the laser diode are mounted on the same moving stage, which is connected to the base attachment via a dovetail translation stage (DT 12, Thorlabs) for focus adjustment. The hemisphere and metal-coated substrates were held together by a sample tray that can be removed from the attachment for loading of new samples and then inserted back (Fig. 1c–f).

After insertion of a sample, the metal side of the substrate faces the smartphone camera, and the glass side faces the hemisphere with an immersion oil applied to fill up the gap (Fig. 1b). During depth of focus adjustments, the laser diode and the sample tray (together with the hemisphere) were moved up and down together to maintain the same illumination angle and location on the sample slide (Fig. 1a). Fluorescent samples under test are placed on the silver side of the substrate with a SiO_2_ spacer layer to prevent fluorescence quenching. The fluorescent signal is collected by an external lens (*f* = 2.6 mm, UCTronics) added in front of the smartphone camera (Nokia 1020). In between the external lens and the mobile phone camera lens, a bandpass filter (525/25 nm, Edmund Optics) is inserted to reject the excitation background. This SEF-based mobile microscope weights approximately 370 grams including the smartphone and the cost of the attachment is estimated to be about $270, excluding the optical filter.

### Optimization of the substrate structure for SEF

In order to find the design with the maximum fluorescence enhancement factor, metal-coated substrates with different silver thicknesses (*t* = 30 or 50 nm) and spacer distances (*d* = 0–50 nm) were prepared. Different incidence angles (*θ*) together with different polarization states (p-pol or s-pol) of the laser beam were tested. These optimization tests were done using a benchtop setup (Supplementary Fig. 1), after which we created a portable mobile phone SEF microscope that is based on the optimal design (Fig. 1).

In the benchtop experimental setup, the laser beam direction was fixed, where the mobile phone and the samples were rotated in order to investigate the angle-dependent fluorescence enhancement effect. To do that, the mobile phone stood vertically at the center of a rotating breadboard (RBB18A, Thorlabs) (Supplementary Fig. 1). Samples (silver-coated substrates together with the hemisphere) were held by a 3D precision stage, which was also immobilized on the same rotating board. The excitation laser (473 nm, 120 mW, Laserglow Technologies) was set on the optical table, pointing to the center of the rotating broad. To find the optimal illumination angle for each substrate design, the stage was rotated with 2-degree increments over a large range of incidence angles (e.g., from 40° to 78°). Mobile phone images of fluorescent objects were then taken at every angular position that is tested.

### Smartphone based imaging of individual 50 nm beads and QDs

Both 50 nm fluorescent beads (Fluoro-Max G50, 468/508 nm) and QDs (Qdot® 625 streptavidin conjugates) were purchased from Thermo Fisher Scientific. Stock fluorescent bead solutions were diluted 10^5^ times in deionized water, and QDs were diluted 10^4^ times in 1x TAE (Tris-acetate-EDTA) buffer, respectively. Small scratches were deliberately made on the metal film as location markers in order to find the same regions on a benchtop microscope that is used for image comparison purposes. 1.5 μL of each diluted solution was then pipetted onto a metal-coated substrate and dried under room temperature. A bandpass filter of 645/75 nm (Olympus) was used for imaging of QDs.

After loading the sample slide onto the hemisphere (immersion oil first applied on the planar surface of the hemisphere) and inserting it into the device, mobile phone images were taken at an exposure time of 4 seconds and ISO 100 setting. Mobile phone focus distance was set to infinity and white balance was fixed for each image acquisition. For the best image contrast, each sample was imaged with multiple frames (e.g., 5–7) in the Digital Negative (DNG) raw format. Image averaging was performed using 16-bit TIFF images converted from DNG format to improve the signal-to-noise ratio of mobile phone images. Benchtop fluorescence microscopy images, used for comparison purposes, were taken with an Olympus IX 73 microscope equipped with a 100× (NA = 1.3) oil immersion objective (UPlanF N, Olympus) and a charge-coupled-device (CCD) based camera (QIClick, QImaging).

### Preparation and smartphone based imaging of the DNA origami brightness standards

Custom built DNA origami nanobeads (GATTA-BEADS) were purchased from GATTAquant. These structures consist of DNA origami nanostructures (average diameter of 23 nm) labelled with a pre-defined number of fluorophores. In order to deposit the DNA origami nanobeads on the silver-coated substrates, first, the stock DNA origami solution (6–10 nM concentration) was diluted 10^3^ times in an imaging buffer. This imaging buffer consisted of 25 mM MgCl_2_ and 4.8% v/v 2-mercaptoethanol (Sigma) in 1x TAE buffer. Cleaned silver-coated substrates were incubated with 0.1% poly-L-lysine (Sigma) for 2 h and dried before the immobilization of DNA origami nanobeads. 1 μL of diluted origami solution was then placed on the metal film, and covered by a 9 × 9 mm coverslip. Nail polish was applied to seal the sample for imaging using our mobile phone microscope. Similar to imaging of QDs, multi-frame capture and averaging, as previously described, were used for imaging these DNA origami samples.

### Simulation of electromagnetic fields on metal-coated films

Simulation of the surface plasmon polariton (SPP) properties of thin metal films was performed using a finite-difference time-domain (FDTD) solution package (Lumerical). Each simulation consisted of perfectly-matched layer (PML) boundaries and 50 nm silver film on a glass substrate and a spacer thickness of 0 to 50 nm with 5 nm step size on top of the silver film, followed by p-polarized (i.e., parallel to the plane of incidence) excitation light with a wavelength of 470 nm, which was launched from glass. An incidence angle sweep of 0 to 75° was performed for each spacer thickness. We then recorded the transmission intensity and the electrical field distribution at the spacer/air interface above the silver film. As for the refractive index values of different materials used in the simulation, we adopted default material properties, as listed in ref. 28.

### Simulation of quantum yield of the fluorophores close to the metal-coated films

In the vicinity of a metal-coated film, fluorophores typically have more pathways to decay from an excited state leading to a reduction of the fluorescence lifetime together with a change in their quantum yield^29^. This change in the quantum yield depends not only on the distance of the fluorophore to the metal film, but also on the relative 3D orientation of the fluorophore with respect to the metal surface, the type of metal employed, the emission band of the fluorophore, and the “intrinsic” quantum yield of the fluorophore in the absence of the metallic film. In most cases, the fluorophore is strongly quenched in close proximity to the metal film with an interaction that weakens with the distance to the film. In order to simulate this effect, we employed a commercially available FDTD software (CST): the fluorophores were modeled using a current source oscillating at their peak emission frequency and a total length of 0.1 nm. The thin film was modeled by a disk with a diameter of 3 µm, i.e., much larger than the maximum thickness of the spacer layer, 50 nm (Supplementary Fig. 2). Since fluorophores bound to the DNA origami structure are free to rotate, both parallel and perpendicular orientations were simulated separately. The average quantum yield of a single fluorophore was calculated by using a weighted average of the parallel and perpendicular orientations, also considering the 2-fold degeneracy of the parallel orientation, i.e., $Average=\frac{2}{3}Parallel+\frac{1}{3}Perpendicular$ ^30^.

## Results and Discussion

### Optimization of the substrate design for maximum fluorescence enhancement

**Figure 2:**
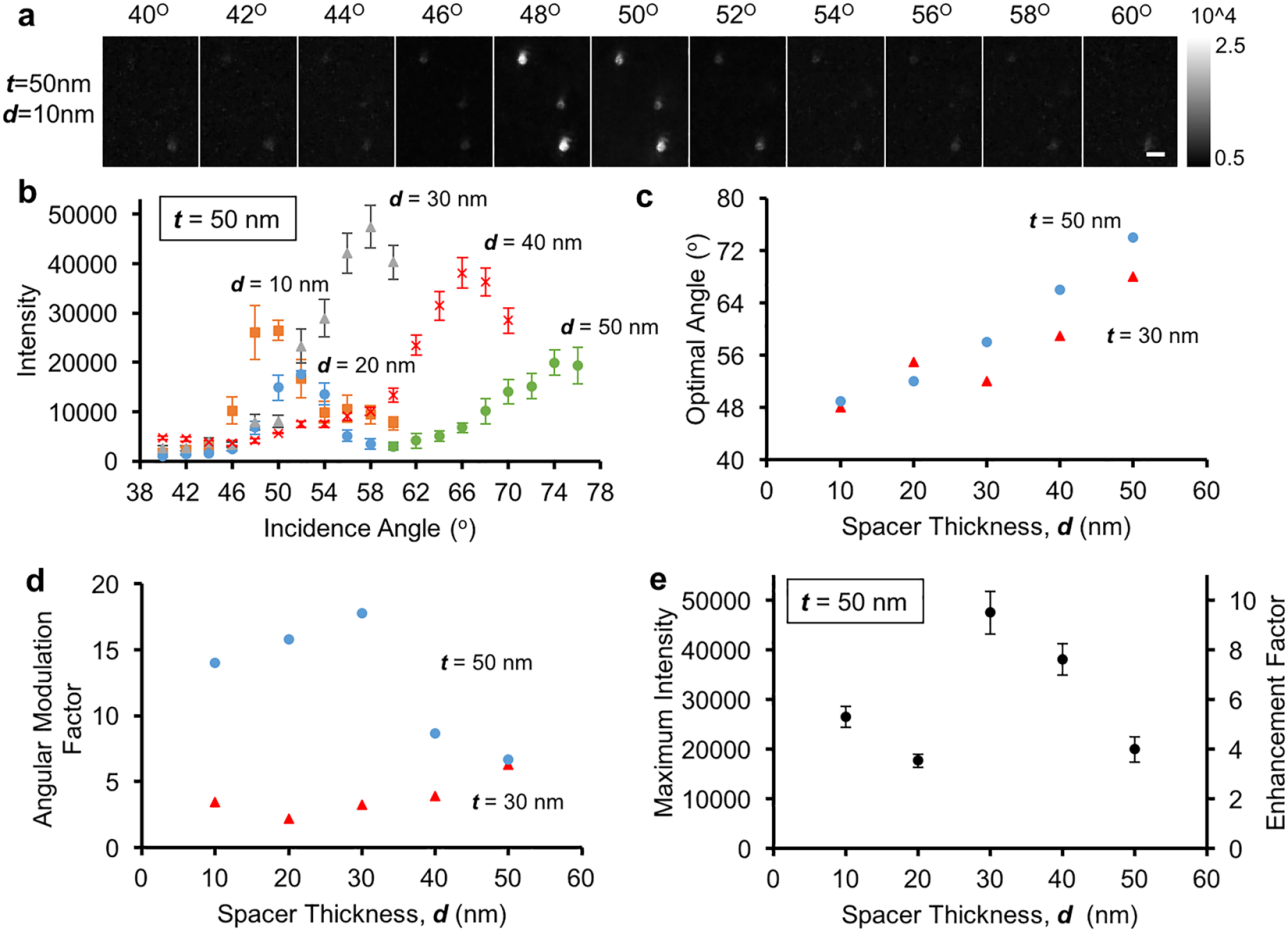
Optimization of the fluorescence enhancement properties of silver thin films. (**a**) Representative angle-dependent mobile phone images of 100 nm fluorescent beads prepared on a 50 nmAg/10 nmSiO_2_ film. Scale bar: 10 μm. (**b**) The effect of the spacer thickness on the fluorescence intensity angular spectra, for the case of 50 nm silver film. (**c**) The relationship of the optimal incidence angle and the spacer thickness. (**d**) The angular modulation factor as a function of the spacer thickness. The excitation angles for different spacer distances correspond to the optimal illumination angles as shown in (**c**). (**e**) Maximum fluorescence intensity for *t* = 50 nm (left y-axis) and the corresponding fluorescence enhancement factor (right y-axis) as a function of the spacer thickness.

SEF phenomenon on a planar thin metal film is strongly dependent on several variables such as the metal thickness (*t*), the spacer distance (*d*), the excitation angle (*θ*), the excitation polarization and wavelength. We systematically optimized the overall performance of our platform by monitoring the angular modulation factor (*AMF*) as well as the enhancement factor (*EF*) of fluorescence due to silver-coated substrates under ~470 nm excitation on a benchtop setup (Supplementary Fig. 1). The excitation wavelength was chosen to approximately match the maximum absorption of silver film and the fluorescent dye for signal enhancement. *AMF* and *EF* for each silver-coated substrate design was calculated using the following equations:

$$AMF=\frac{Flurescence\,Intensity\,at\,the\,Optimal\,Angle\,(Silver\,Film)}{Flurescence\,Intensity\,at\,the\,Base\,Line\,(Silver\,Film)}$$

$$EF=\frac{Flurescence\,Intensity\,at\,the\,Optimal\,Angle\,(Silver\,Film)}{Flurescence\,Intensity\,at\,the\,Optimal\,Angle\,(Base\,Glass)}$$

where *AMF* is obtained by dividing the average fluorescence intensity of target nanoparticles at the optimal angle (yielding maximum fluorescence) by their base line intensity in the angular intensity spectrum of a given plasmonic substrate design (such as Fig. 2b). *EF*, on the other hand, is obtained by dividing the average fluorescence intensity of nanoparticles on silver films at the optimal illumination angle with the average fluorescence intensity that can be obtained on a bare glass using an optimal illumination angle (~75°, as reported in our earlier work^26, 27^). In these optimization experiments, fluorescent beads with 100 nm diameter (Fluoro-Max G100, 468/508 nm Thermo Fisher Scientific) were deposited on 10 different designs of silver-coated substrates (*t* = 30 nm, *d* = 10, 20, 30, 40, 50 nm; *t* = 50 nm, *d* = 10, 20, 30, 40, 50 nm) and imaged using our mobile phone based system with 20 different incidence angles (*θ* = 40–78°, Δ*θ* = 2°). At each imaging configuration, more than 100 individual particles were measured and their intensities were averaged to form an angular intensity spectrum for each substrate design (see e.g., Fig. 2b). As expected, p-polarized light more effectively excites SPP on the surface of the thin metal film, also evidenced by the higher *AMF* values measured with p-pol excitation compared to s-pol for different spacer thickness values on 50 nm thick silver film (see Supplementary Fig. 3). All the subsequent measurements were therefore based on p-polarized excitation light.

Spacer distance and angle of illumination also play significant roles in tuning the performance of our mobile SEF microscope. Figure 2a shows a series of angle-dependent images captured by the mobile phone microscope using a 50 nmAg/10 nmSiO_2_ substrate design. The background of each image was normalized to provide a fair comparison and the brightest particle intensities for this substrate design (*t* = 50 nm, *d* = 10 nm) were observed at an incidence angle of around 48°. Similar analysis was also performed for all the other silver-coated substrate designs, and the angular dependence of our results corresponding to 50 nm silver films are summarized in Fig. 2b. Clearly, the optimal angle that produces the maximum fluorescence enhancement factor is highly dependent on the spacer thickness (Fig. 2b). The optimal incidence angle increases with the increase of the spacer thickness, and a similar trend is observed for both 50 nm and 30 nm silver films (Fig. 2c).

The trend of *AMF* as a function of the spacer thickness is also plotted in Fig. 2d. For *t* = 50 nm silver film, *AMF* increases with increasing spacer distance until reaching a maximum value of ~18 at *d* = 30 nm, and then decreases as the spacer is further increased (Fig. 2d). For *t* = 30 nm silver film, on the other hand, the *AMF* values are in general much smaller than 50 nm silver-coated substrate designs (Fig. 2d). The maximum fluorescence intensities without the angular normalization step (as used in *AMF*) are subject to increased experimental variation (see Fig. 2e, left y-axis). However, despite this experimental variation, a peak fluorescence intensity was clearly observed with a spacer thickness of ***d*** = 30 nm (Fig. 2e). Moreover, compared to the optimal imaging conditions for a bare glass substrate, a fluorescence *EF* of ~9.5 was observed for the 50 nmAg/30 nmSiO_2_ substrate design (Fig. 2e, right y-axis).

**Figure 3:**
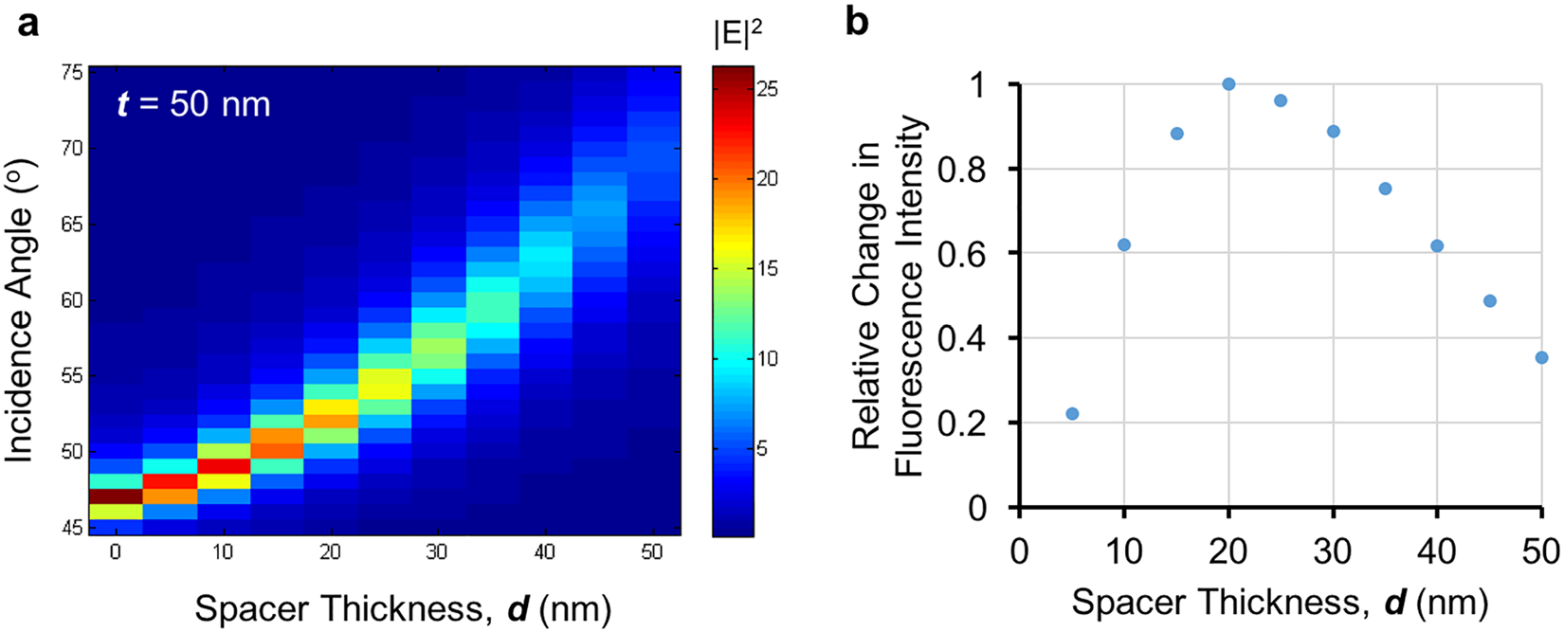
Numerical simulation of surface-enhanced fluorescence in close proximity of a thin silver film. (**a**) Numerical simulation of the electric field intensity at the SiO_2_-air interface, corresponding to 50 nm silver-coated substrates as a function of the incidence angle and the spacer distance under 470 nm illumination. (**b**) Simulated relative change in fluorescence intensity of a fluorophore in the vicinity of a 50 nm thick silver film as a function of the spacer distance.

As illustrated in these results, *EF* values are found to be very sensitive to the spacer thickness, ***d***. This can be attributed to the trade-off between fluorescence enhancement and quenching phenomena when placing a fluorophore in close proximity to a metal surface. Numerical simulations were also conducted to validate these experimental observations. First, the electromagnetic field intensity at the SiO_2_ spacer-air interface, corresponding to 50 nm silver-coated substrates was simulated as a function of the illumination angle and the spacer distance when exposed to 470 nm excitation. As shown in Fig. 3a, the optimal incidence angle also increases with the increase in spacer thickness, matching well with our experimental results shown in Fig. 2c. Moreover, our numerical simulations for *t* = 50 nm suggests that the field intensity rapidly decays as a function of the increasing spacer distance (Fig. 3a), leading to a quick loss of field enhancement effect when the distance between a fluorophore and metal surface increases. Second, the fluorescence quantum yield of a fluorophore was simulated by assuming that a fluorophore can be represented as a randomly oriented dipole, which is placed at various different spacer distances on top of a 3 μm diameter and *t* = 50 nm thick silver disk to mimic a larger planar thin film (Supplementary Fig. 2). Our simulation results show that significant fluorescence quenching is observed for spacer distances smaller than 20 nm, where the parallel dipole orientation is quenched even more rapidly than the perpendicular dipole orientation (Supplementary Fig. 2b). Based on the product of the field intensity at the SiO_2_-air interface and fluorophore quantum yield, we obtained the simulated relative change in fluorescence intensity as a function of the spacer distance, as shown in Fig. 3b. The results show a trend of first increasing and then decreasing fluorescence intensity when the spacer thickness increases (Fig. 3b), also matching well with our experimental observations for *t* = 50 nm (Fig. 2d). However, the numerically predicted maximum fluorescence enhancement location or SiO_2_ layer thickness (*d* = 20 nm in Fig. 3b) is slightly different from our experimental results for *t* = 50 nm, which predicted *d* = 30 nm as the optimal spacer thickness. Various effects may be responsible for this mismatch, including the averaging between different fluorophore-film orientations, the intrinsic photochemical properties of the fluorophores as well as the optical properties of the silver film.

The above summarized substrate optimization experiments, well agreeing with our numerical simulations, suggest that a maximum fluorescence enhancement factor of ~10 can be achieved on a silver-coated substrate design with *t* = 50 nm and *d* = 30 nm, and 58° incidence angle when excited at ~470 nm light. This substrate design provides a unique opportunity for approximately an order of magnitude fluorescence intensity improvement compared to the use of conventional glass substrates. Although much stronger enhancement factors (e.g., ~1000 fold) have been reported for single-molecule imaging using more advanced plasmonic substrates based on e.g., nano-antenna structures^31–33^, the presented thin metal film based design has its own advantages such as being extremely easy to fabricate, cost-effective to scale up, uniform in its enhancement field, and simple for sample preparation and alignment. All of these unique features make thin metal film based substrates quite promising for future POC diagnostic applications based on molecular imaging and sensing.

### Fluorescence imaging of individual 50 nm beads and QDs using a smartphone-based SEF microscope

**Figure 4:**
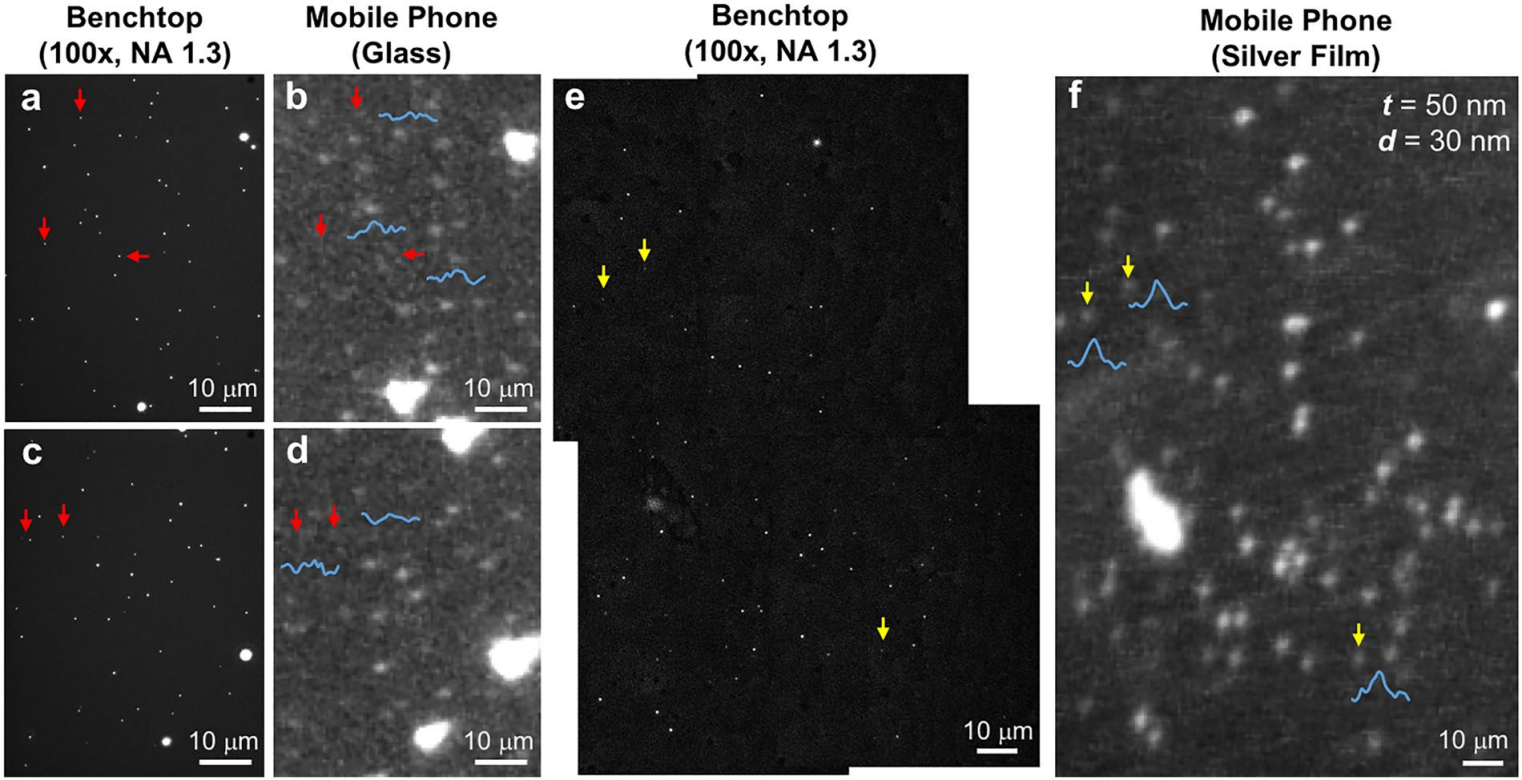
Imaging of individual 50 nm fluorescent beads using the smartphone SEF microscope. (**a**–**d**) Control experiments by imaging the 50 nm fluorescent bead samples with a benchtop microscope (**a**,**c**) and a previous mobile phone microscopy device using glass substrates (**b**,**d**), respectively. Some examples of missed 50 nm particles by the glass-based mobile phone imaging device are highlighted by red arrows, and the corresponding intensity line scans shown in blue curves. (**e**,**f**) Detection of individual 50 nm particles by the newly developed mobile phone SEF imaging device (**f**); the same field-of-view was also imaged by a conventional benchtop microscope (**e**), 4 images stitched together. Yellow arrows indicate some examples of the detected 50 nm particles and blue curves show their corresponding intensity line scans.

Our smartphone based SEF microscope (Fig. 1) was prepared based on the best performing silver-coated substrate design (i.e., 50 nmAg/30 nmSiO_2_) as a result of the above described optimization experiments. This mobile microscope was then used for all the subsequent imaging studies. We first imaged 50 nm fluorescent particles (Fig. 4 and Supplementary Fig. 4). These samples were imaged by two different mobile phone microscope platforms for direct comparison: (1) a mobile phone based fluorescence microscope that is built based on the same smartphone, laser diode and filter set, but using standard glass substrates and, (2) our newly developed mobile phone based SEF microscope using silver-coated substrates. A benchtop fluorescence microscope with an oil-immersion objective lens (NA = 1.3) was used as gold standard to validate our mobile phone imaging results. As shown in Fig. 4a–d, the mobile phone fluorescence microscope with glass-based substrates missed many of the individual 50 nm fluorescent beads, as also highlighted by the red arrows and the corresponding intensity profile scans reported in Fig. 4b,d. In contrast, individual 50 nm beads were all successfully detected by the smartphone based SEF microscope (Fig. 4e,f), and a very good signal intensity and image contrast were observed for all the detected 50 nm particles, as highlighted by the yellow arrows and the corresponding intensity profile scans reported in Fig. 4f.

**Figure 5:**
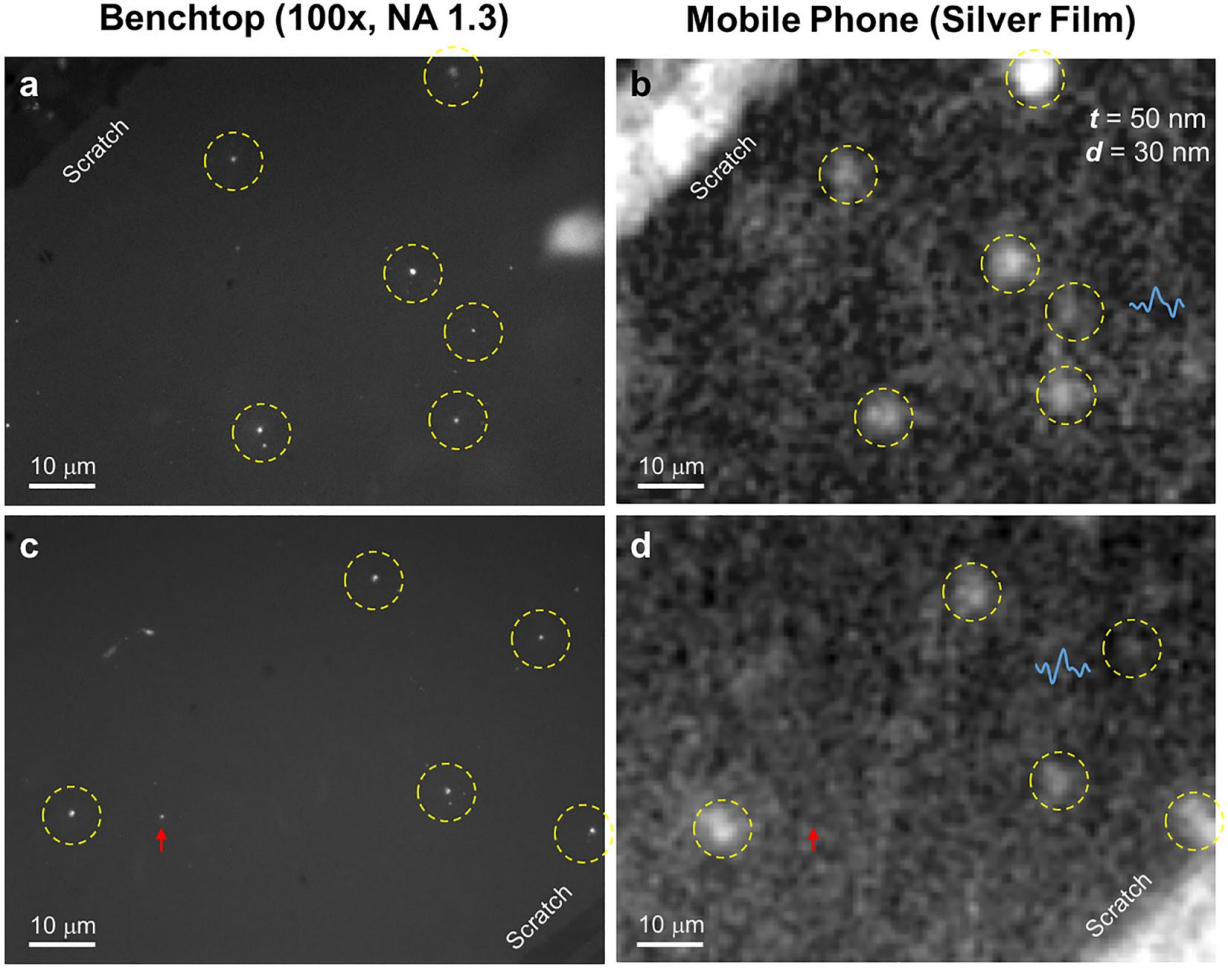
Imaging of single QDs with our smartphone-based SEF microscope. (**a**,**c**) Single QDs imaged by a conventional benchtop microscope. (**b**,**d**) Same regions of interest imaged by the mobile SEF microscope, where the yellow dashed circles highlight the successful detection of single QDs on the mobile phone, and the red arrow represents a missed detection. Two intensity line scans, corresponding to weaker QDs, are also shown (blue curves).

Next, we imaged diluted QD samples using the same smartphone SEF microscope. Figure 5 exhibits two representative regions of interest of the same QD sample imaged by both a conventional benchtop microscope (100×/NA = 1.3) and our smartphone-based SEF microscope. The presence of each individual QD was confirmed by the corresponding images of the benchtop microscope and highlighted by yellow dashed circles (Fig. 5a,c). Except a single case, all the isolated QDs in the same samples were also detected in our mobile phone images (Fig. 5b,d). The red arrow in Fig. 5d shows the failed QD detection on the mobile phone image, plausibly due to the fluorescence intermittency and blinking behavior of single QDs, especially if they are switched to dark states during the mobile phone image acquisition period, or at least part of it.

### Quantification of the detection limit of smartphone-based SEF microscopy

**Figure 6:**
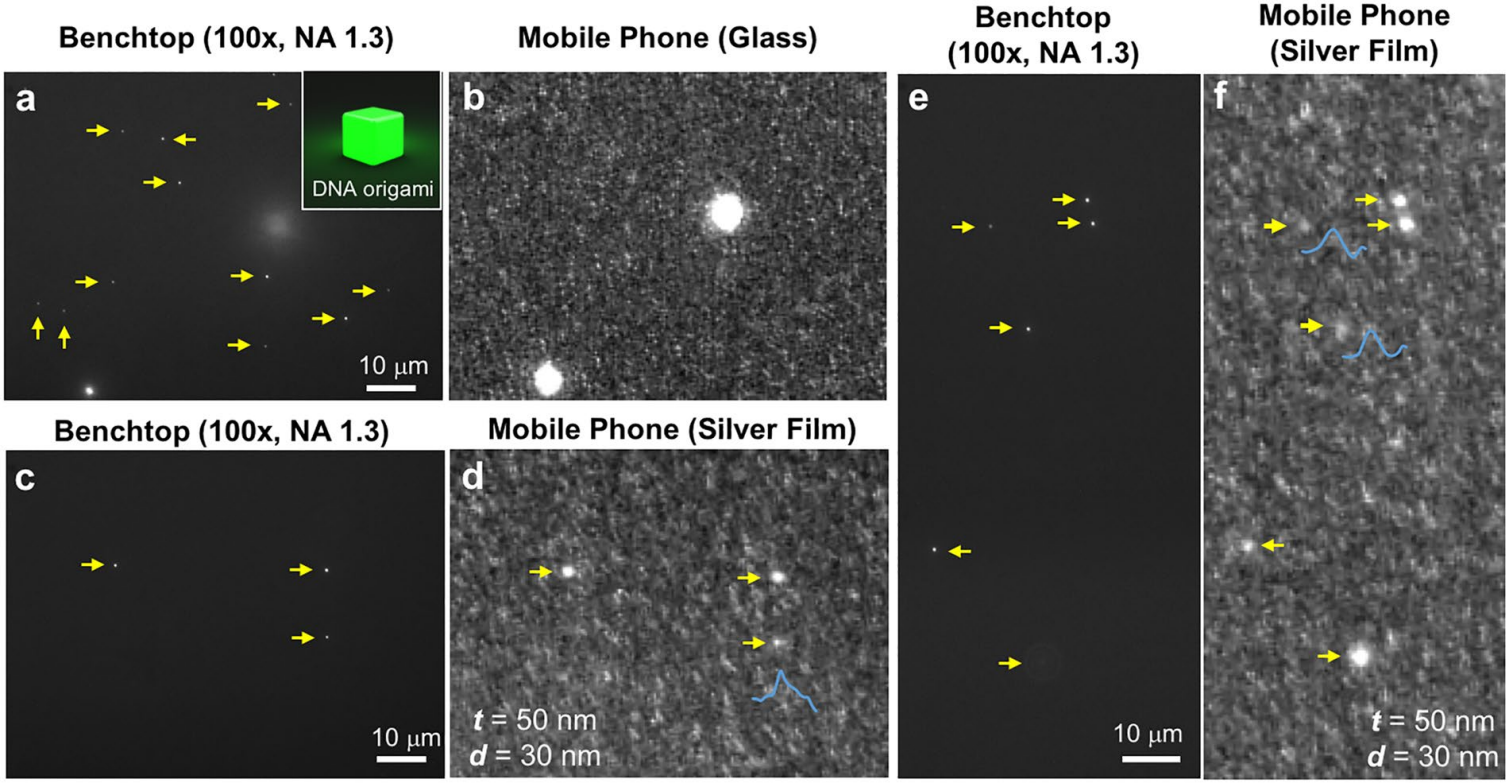
Imaging of single DNA origami nanobeads labeled with 80 ± 4 fluorophores, each. (**a**,**b**) Control experiments for imaging the DNA origami nanobeads using a glass-based mobile phone microscope (**b**), and the same region of interest imaged by a benchtop microscope (**a**). A schematic illustration of a fluorescent DNA origami nanobead is shown in the inset of (**a**). (**c**–**f**) Detection of single 80-fluorophore DNA origami nanobeads with our smartphone-based SEF microscope. The sample was imaged by both a benchtop microscope (**c**,**e**) and our smartphone-based SEF microscope (**d**,**f**) to validate the detection of single DNA origami nanobeads. DNA origami nanobeads are highlighted by the yellow arrows and the intensity line scans of three weaker ones are also shown (blue curves). In (**e**) the bottom yellow arrow points to an out-of-focus fluorescent origami structure.

To quantify the detection limit of our smartphone-based SEF microscope, 23 nm diameter DNA origami structures labeled with different numbers of fluorophores were used as test samples and imaged by our mobile phone SEF microscope. Being highly programmable, DNA origami structures have precisely controlled shapes and available binding sites. As a result, the number of labeled fluorophores per origami structure can also be fine-tuned, making DNA origami nanobeads a standard for quantification of fluorescence nanoscopy and single-molecule microscopy techniques^34, 35^. We tested our smartphone SEF microscope with DNA origami nanobeads (inset of Fig. 6a) labeled with 80 ± 4, 42 ± 10, and 25 ± 16 Alexa488 fluorophores per origami structure, respectively. As expected, the fluorescence intensity of each DNA origami structure increased with the increase in the number of fluorescent molecules that is loaded per structure, which is also confirmed by using a benchtop fluorescence microscope with an oil-immersion objective (NA = 1.3) (Supplementary Fig. 5). The mean fluorescence intensity of 42 ± 10 fluorophores per origami is approximately 50% weaker than that of the 80 ± 4 fluorophore sample, see Supplementary Fig. 6.

Control experiments were conducted by using standard glass-based substrates to image DNA origami nanobeads on a mobile phone based fluorescence microscope that is built based on the same smartphone, laser diode and filter. 80-fluorophore DNA origami nanobeads were mixed with 100 nm green fluorescent beads, and only the latter could be detected using glass-based substrates (Fig. 6a,b). On the other hand, our smartphone-based SEF microscope was able to image individual DNA origami nanobeads based on the enhancement provided by silver-coated substrates (*t* = 50 nm, *d* = 30 nm). Figure 6c,d,e,f show two different regions of interest of the same sample imaged by both a benchtop microscope and our mobile phone SEF microscope. Clearly, each 80-fluorophore DNA origami nanobead that is detectable by the 100× oil-immersion objective on a conventional benchtop microscope (Fig. 6c,e) can also be detected using our mobile phone microscope (Fig. 6d,f). Even the weaker DNA origami nanobeads in Fig. 6c,e displayed quite strong signal-to-background contrast in our mobile phone SEF images as shown by their corresponding intensity profiles (blue curves in Fig. 6d,f).

**Figure 7:**
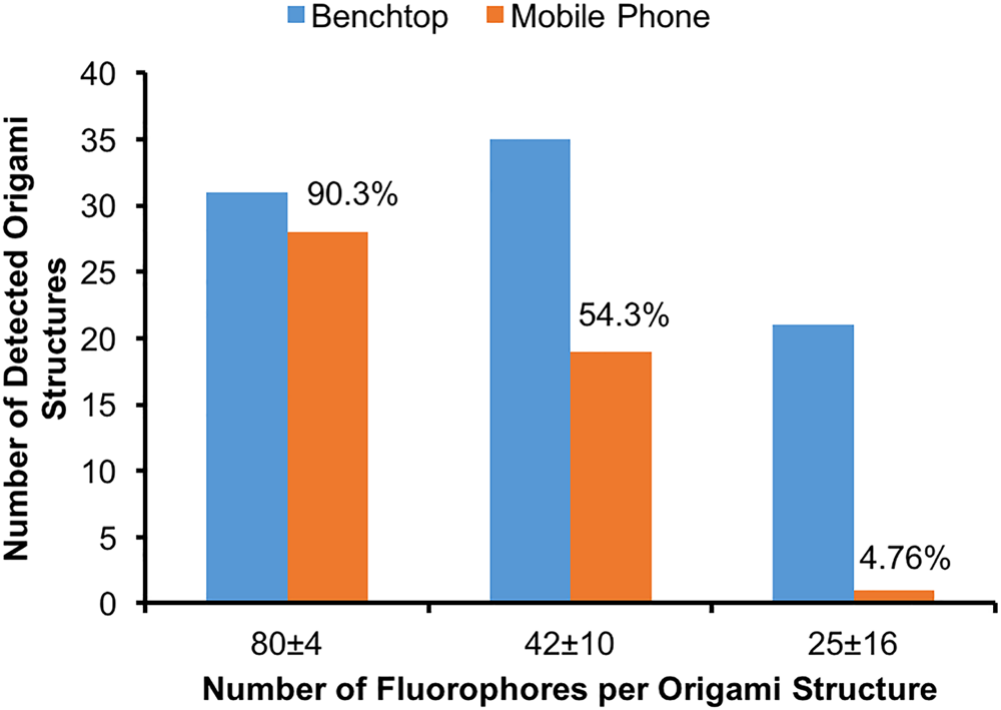
Detection efficiency of different DNA origami nanobeads labeled with 80 ± 4, 42 ± 10, and 25 ± 16 fluorophores by the smartphone SEF microscope. The detection efficiency was calculated by dividing the number of detected origami nanobeads using the mobile phone SEF microscope by the total number that is detected with a benchtop microscope over the same region of interests.

DNA origami nanobeads with even lower fluorophore numbers were also tested by our smartphone SEF microscope to assess the limit of detection. As shown in Fig. 7, we were able to detect ~90.3% of 80-fluorophore DNA origami nanobeads when compared to the benchtop microscope results, used as gold standard, while the detection rate of our mobile phone microscope decreased to ~54% and ~5% for 42- and 25-fluorophore DNA origami nanobeads, respectively. Less than 100% detection efficiency of 80-fluorophore DNA origami nanobeads using our smartphone SEF microscope can be attributed to the fabrication variations observed in each batch of DNA origami structures, as also highlighted in Supplementary Fig. 6. Therefore, the limit of detection of our smartphone SEF microscope is estimated to be approximately 80 fluorescent molecules per diffraction limited spot, which provides an order of magnitude improvement compared to previous mobile phone fluorescence microscopes using only glass-based substrates.

To summarize, in this work we showed that nanophotonic solutions on the substrate design can leverage handheld devices such as mobile phone microscopes towards ultra-sensitive fluorescence detection using a cost-effective and field portable interface. Using easy-to-fabricate silver-coated glass substrates, we achieved ~10 fold fluorescence intensity enhancement compared to conventional bare glass substrates, which enabled us to detect individual 50 nm fluorescent particles as well as individual QDs using a mobile phone microscope. We also confirmed that the same SEF-based mobile phone microscope can routinely detect ~80 fluorophores per diffraction-limited spot, opening up various opportunities for POC diagnostics and molecular sensing applications in resource scarce settings. We see these presented results as an important step forward to engineer consumer electronics based detection devices for ultra-sensitive analysis using nanophotonic designs.

## Electronic supplementary material

Supplementary Information
