# Supplementary material for: Plasmonics Enhanced Smartphone Fluorescence Microscopy
Source: Sci Rep. 2017 May 18;7:2124. doi: 10.1038/s41598-017-02395-8 (PMC5437072; doi:10.1038/s41598-017-02395-8)
Supplement: Supplementary file 1 — Supplementary Information [file 41598_2017_2395_MOESM1_ESM.pdf]

Supplementary Information

Plasmonics Enhanced Smartphone Fluorescence Microscopy

Qingshan Wei,[1,2,3,4,†] Guillermo Acuna,[5,6,7,†] Seungkyeum Kim,[8,†] Carolin Vietz,[5,6,7] Derek Tseng,[1] Jongjae Chae,[1] Daniel Shir,[1] Wei Luo,[1] Philip Tinnefeld,[5,6,7,*] and Aydogan Ozcan[1,2,3,9,*]

1. Electrical Engineering Department, University of California, Los Angeles, Los Angeles, CA, 90095, USA
2. Bioengineering Department, University of California, Los Angeles, Los Angeles, CA, 90095, USA
3. California NanoSystems Institute (CNSI), University of California, Los Angeles, Los Angeles, CA, 90095, USA
4. Department of Chemical and Biomolecular Engineering, North Carolina State University, Raleigh, NC, 27695, USA
5. Institute for Physical & Theoretical Chemistry, Braunschweig University of Technology, Braunschweig 38106, Germany
6. Braunschweig Integrated Centre of Systems Biology (BRICS), Braunschweig University of Technology, Braunschweig 38106, Germany
7. Laboratory for Emerging Nanometrology (LENA), Braunschweig University of Technology, Braunschweig 38106, Germany
8. Chemical and Biomolecular Engineering Department, University of California, Los Angeles, Los Angeles, CA, 90095, USA
9. Department of Surgery, University of California, Los Angeles, Los Angeles, CA, 90095, USA

† Joint first authors
* Corresponding authors: p.tinnefeld@tu-braunschweig.de ; ozcan@ucla.edu

Supplementary Figures:

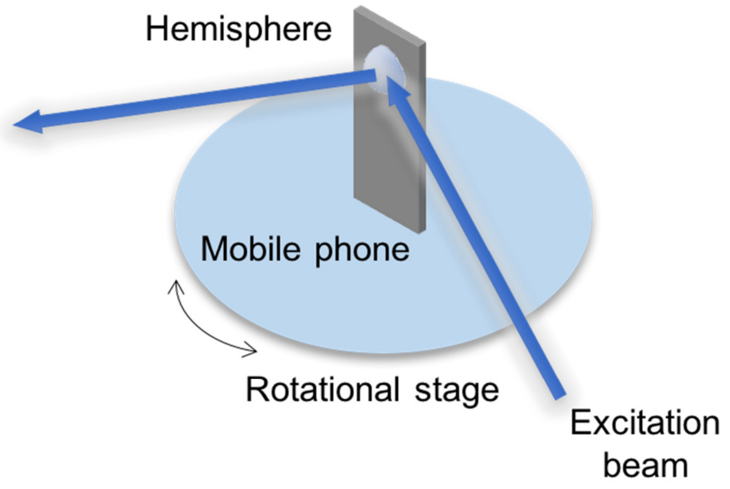

Supplementary Figure 1. Schematic of the benchtop setup for the optimization of silver substrates using a rotational stage.

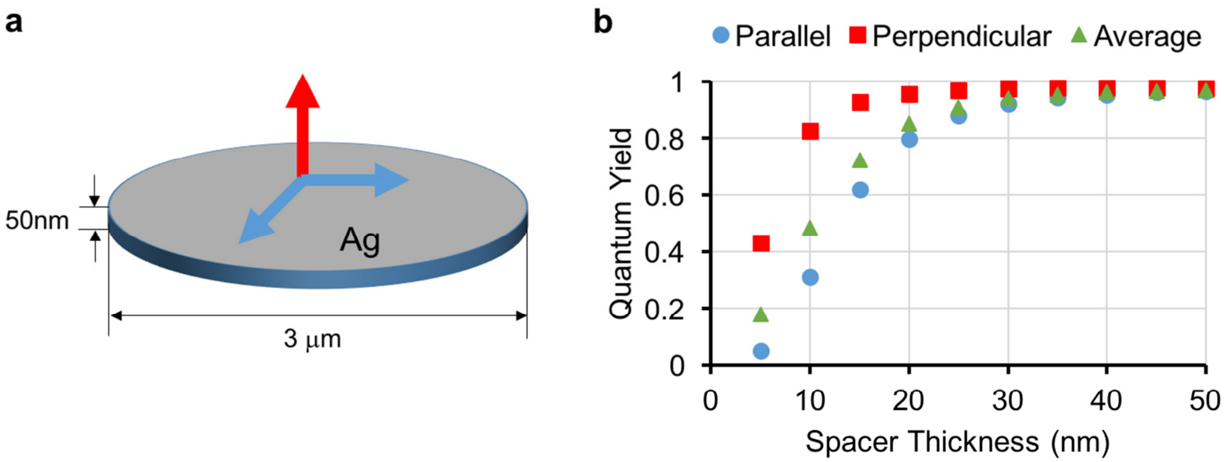

Supplementary Figure 2. Simulation of fluorescence quantum yield in close proximity of a silver film. (a) Schematic illumination of two typical orientations (red: perpendicular and blue: parallel) of a fluorophore dipole placed on a planar silver film. (b) Simulated quantum yield of the fluorophore in the vicinity of the 50 nm silver film. The average quantum yield is calculated by using the equation: Average = (Parallel)*2/3 + (Perpendicular)*1/3.

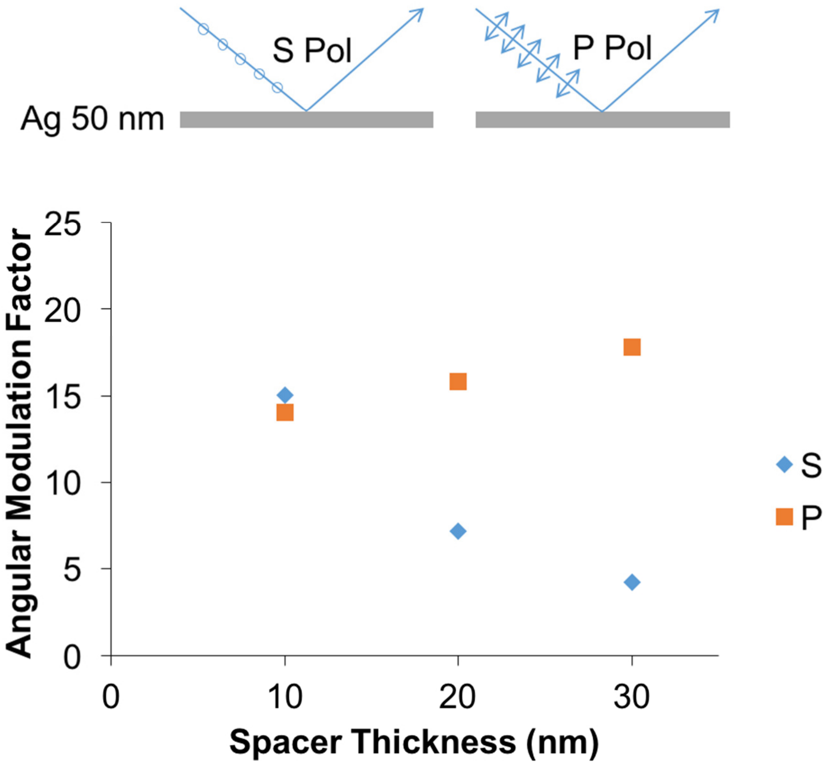

Supplementary Figure 3. Effect of the polarization of the excitation source on the surface enhancement properties of 50 nm silver thin films. Two different polarization states (s, and p-pol) are illustrated in the inset, and the angular modulation factor is plotted as a function of the spacer thickness under each polarization.

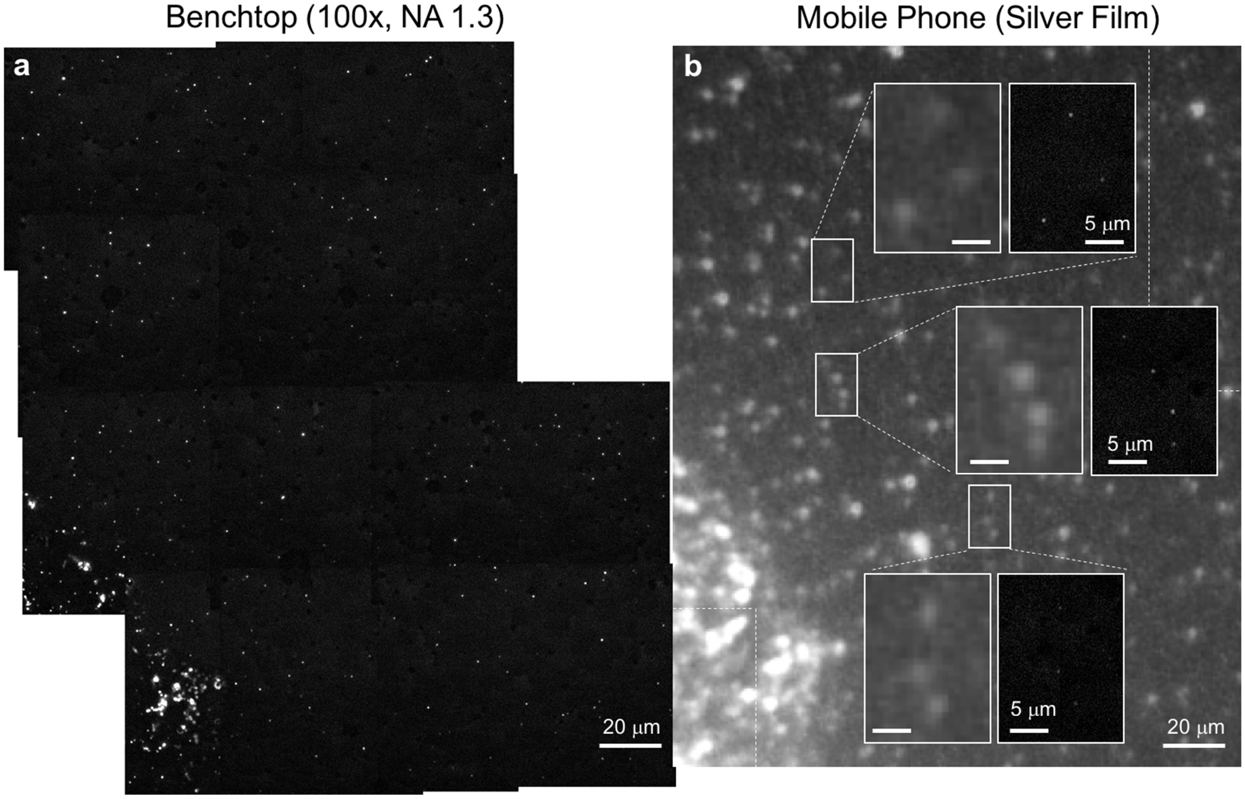

Supplementary Figure 4. Imaging of single 50 nm fluorescent beads using the smartphone-based SEF microscope. (a) 50 nm fluorescent beads imaged by a benchtop microscope (stitched image, composed of 12 individual scans). (b) The same region of interest imaged by the mobile phone SEF microscope using the optimized silver substrate (***t*** = 50 nm, ***d*** = 30 nm). Three insets show zoomed-in regions of the mobile phone images and the corresponding microscope comparison images, verifying the detection of every single 50 nm bead using the mobile phone microscope.

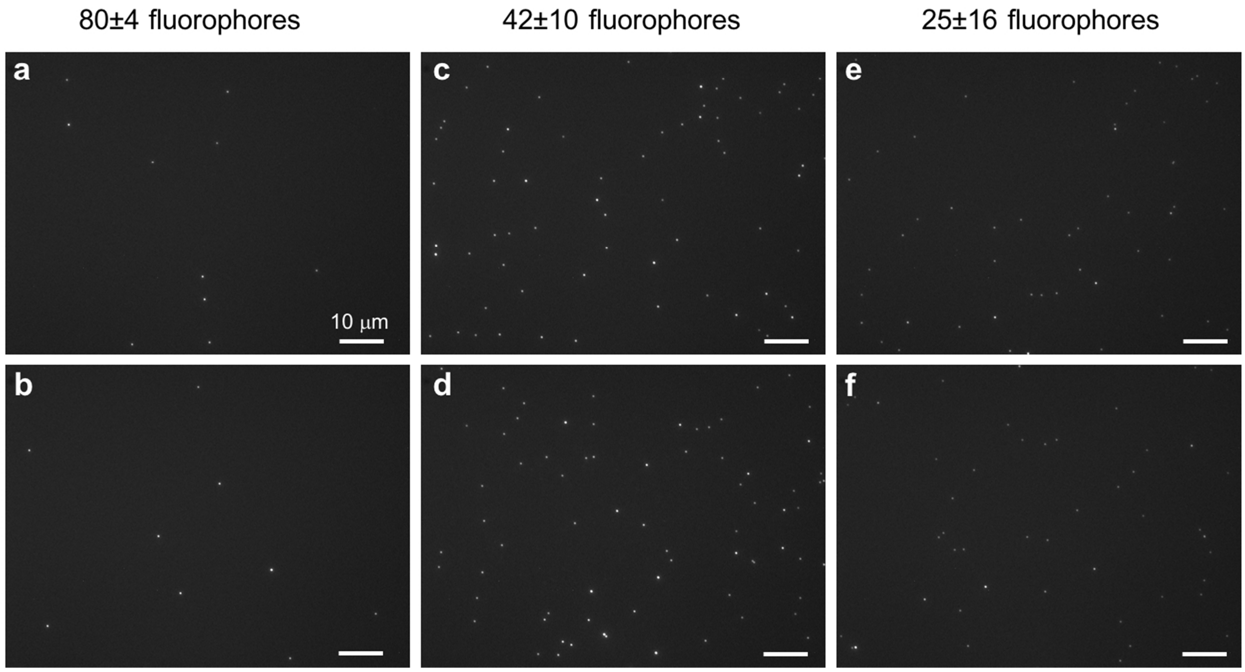

Supplementary Figure 5. Benchtop fluorescence microscope images of three different DNA origami structures used for testing the detection limit of the mobile SEF imaging devices. The DNA origami beads were labeled with 80±4 (a,b), 42±10 (c,d), and 25±16 (e,f) fluorophores, respectively.

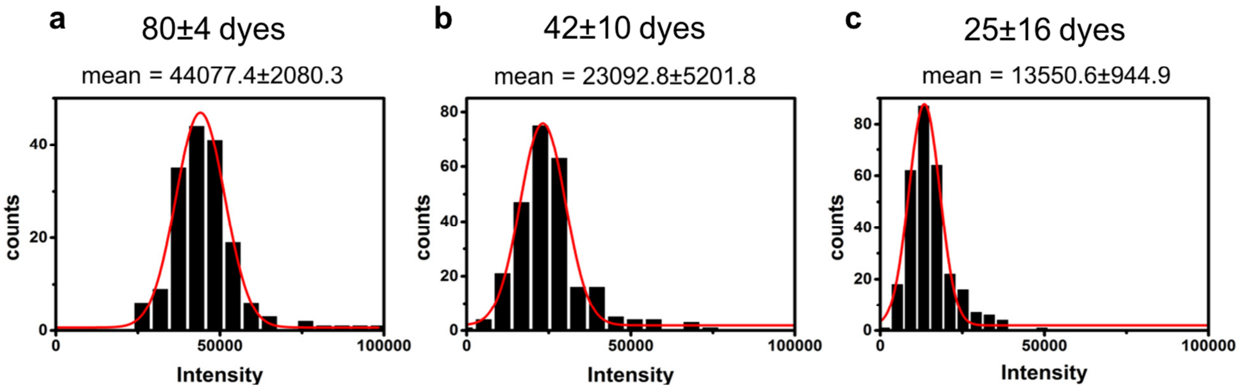

Supplementary Figure 6. Intensity histograms of fluorescent DNA origami structures measured by a benchtop fluorescence microscope: (a) 80-fluorophores, (b) 42-fluorophores, and (c) 25-fluorophores, respectively.
